# Breastfeeding practice and factors associated with exclusive breastfeeding among mothers in Horro District, Ethiopia: A community-based cross-sectional study

**DOI:** 10.1371/journal.pone.0267269

**Published:** 2022-04-27

**Authors:** Debela Daba Jebena, Mesfin Wogayehu Tenagashaw

**Affiliations:** Department of Applied Human Nutrition, Bahir Dar Institute of Technology, Bahir Dar, Ethiopia; Texas A&M University College Station, UNITED STATES

## Abstract

**Background:**

Breastfeeding is the process of feeding a newborn with the mother’s milk, and it is very important for enhancing child and maternal health. The proportion and duration of breastfeeding may vary by location, and is poorly practiced for cultural, economic, and societal reasons. Thus, this study was conducted to determine breastfeeding practices and determinants of exclusive breastfeeding among mothers of six month aged infants in the Horro district, Ethiopia.

**Methods:**

We used a community-based cross-sectional study among 649 mothers of index infants. A multi-stage sampling procedure was used to select the women. Data were collected from March 15 to April 5, 2020. Face-to-face interviews were used to collect data using a semi-structured questionnaire. Bivariable and multivariable logistic regression were performed to examine the factors associated with exclusive breastfeeding.

**Results:**

All women who participated in the study have ever breastfed their children at some point. Exclusive breastfeeding and breastfeeding initiation were found to be good (70.4% and 61.8%, respectively) within 24 hours prior to the survey time. Having had information about breastfeeding during antenatal care (AOR = 4.15, 95% CI = 2.36, 7.30), postnatal care follow-up (AOR = 4.74, 95% CI = 2.92, 7.70), having infant aged 0-1month (AOR = 12.14, 95% CI = 3.83, 38.46) and 2–3 month (AOR = 8.62, 95% CI = 5.00, 14.85), being a single birth (AOR = 12.91, 95% CI = 3.86, 43.21), having monthly income of ≤ 100 Ethiopian Birrs (AOR = 1.96, 95% CI = 1.16, 3.32), and breastfeeding initiation within one hour of birth (AOR = 1.94, 95% CI = 1.13, 3.35) were found to be a significantly associated factors of exclusive breastfeeding.

**Conclusion:**

Despite meeting the global nutrition target of 2025, the practice of exclusive breastfeeding was lower than the WHO recommendations. Providing education about breastfeeding during antenatal care follow-up and increasing access to postnatal care follow-up is recommended to enhance exclusive breastfeeding practices in the study area.

## Backgrounds

Malnutrition is the biggest cause of death and illness in the world, outnumbering the costs of several other important global health issues. In today’s world, one out of every nine people is hungry, while one out of every three is overweight or obese [1]. In many countries, undernutrition coexists with overweight, obesity, or other diet-related non-communicable diseases, resulting in a double burden of malnutrition [1, 2]. Undernutrition is responsible for 2.7 million child deaths per year, which accounts for 45% of all child mortality [3].

According to the convention on the rights of the child, every infant and child has the right to appropriate nutrition [4]. Breastfeeding (BF) is one of the most important factors for the health, development, and survival of a child. The World Health Organization (WHO) recommends initiating breastfeeding within the first hour of birth and exclusively breastfeeding for the first six months. Complementary feeding should be introduced after six months, while breastfeeding is expected to continue until the child is 24 months old or older [5]. According to WHO, “*exclusive breastfeeding (EBF) is defined as an infant receiving only breast milk from the mother or a wet nurse*, *or expressed breast milk*, *and no other liquids or solids*, *including water*, *with the exception of oral re-hydration solution*, *drops or syrups containing vitamins*, *mineral supplements*, *or medicines”* [3, 6].

Breastfeeding is an important method for improving public health as it has numerous documented health advantages for babies and mothers [7]. For babies, it offers irreplaceable nutrients for growth and development, decreases the risk of becoming overweight or obese, and protects against certain non-communicable diseases later in life, helps with brain and nervous system development, as well as having higher intelligence quotients (IQs) [8, 9]. It can also protect the child from respiratory infections, diarrheal sickness, and other potentially life-threatening illnesses [9]. Allergies, colds, diaper rashes, and ear and stomach disorders are less common in breastfed babies [8, 9]. For the mother, it helps the uterus contract to return to its normal size [10], and it creates strong bonding between a mother and a baby [8]. Breastfeeding for longer periods of time benefits mothers’ health and well-being by lowering the risk of ovarian and breast cancer and spacing pregnancies [11]. Breastfeeding is convenient and costs less than formula [12].

Despite mounting evidence that non-breastfeeding is linked to higher mortality, significant morbidity, and other long-term negative health consequences, governmental attempts to enhance EBF and sustain breastfeeding rates have had only a minor impact. Globally, 41% of infants aged less than six months were exclusively breastfed in 2017, and only 45% continued BF until two years old [5]. Many African mothers breastfeed their newborns for more than a year, but EBF for up to six months is relatively uncommon [13]. The Ethiopian Demographic and Health Survey (EDHS) 2016 revealed that almost all children (97%) are breastfed at some point, and only 58% of infants under the age of six months are breastfed exclusively. Many newborns are fed other liquids such as water (17%), non-milk liquids (5%), and other milks (5%) before they reach the age of six months [14]. Inadequate rates of EBF are due to societal and cultural reasons, lack of awareness about nursing, lack of partner’s support and encouragement, as well as other support personnel [8, 9]. Furthermore, the associated factors vary among countries and even within a country [15]. For these reasons, it is recommended that each country create infant and early childhood nutrition surveillance as an integral part of its health information system. Breastfeeding patterns and feeding practices in newborns and young children should be observed on a regular basis in order to identify difficulties and establish solutions to prevent illness and poor growth [16]. The study examined breastfeeding practices and factors associated with EBF among mothers in the Horro district, Ethiopia.

## Methods

### Study design and setting

This cross-sectional survey was carried out in Horro district, which is located in Horro Guduru Wollega Zone, Oromia Regional State, Ethiopia, around 315 kilometers west of the country’s capital, Addis Ababa. This district has an elevation of 2503 meter above sea level and located at 9° 34′ 0″ North, 37° 6′ 0″ East. The district consists of both rural and urban residents. A majority of the people are engaged in agricultural activities as a major livelihood means. The district has one hospital, seven health centers and 23 health posts owned by the government and five privately owned clinics. The study was conducted from March 15 to April 5, 2020.

### Sample size

The sample size was calculated using a single population proportion formula. The following assumptions have been made: a 95% confidence level, a 5% margin of error, and the prevalence of EBF practice (P = 43.6%) at Bedele [17], a finite population correction factor formula since the total number of under 6 month lactating mothers in the district (N) was 1332 according to the data from the district health office, a 10% non-response rate, and a design effect of 2 for the multi-stage sampling. The final sample size was 649 mothers.

### Sampling technique

There were 23 Kebeles (i.e., the smallest unit of administration) in the Horro district, two of which were urban and twenty-one of which were rural. Thirteen kebeles (1 urban and 12 rural) were selected using simple random sampling, a lottery method. The number of study participants was proportionally distributed to each Kebele depending on the number of mother-infant pairs in each kebele.

As a sampling frame, the local health extension worker’s registration form for women with infants under the age of six months was used. In order to obtain the required sample size as per the proportional allocation, mothers who had an infant aged less than 6 months were selected by employing simple random sampling techniques. Only mothers who are permanent residents of the selected kebeles were included.

### Data collection tools, procedures and quality control

Semi-structured questionnaire was prepared by adopting from the EDHS (2016) and other related studies which were designed to assess infant and young child feeding practices in developing countries [14, 18–20]. The questionnaire considered the study objectives and the local situation of the study area. It was initially prepared in English and then translated into Afaan Oromo, the local language, and translated back to English by language experts to ensure consistency. The questionnaire was pre-tested on 5% of the sample size prior to the real data collection process at non-sampled Kebeles, and modifications were made as needed. Face-to-face interviews were conducted to collect data on socio-demographic variables, obstetric characteristics, and EBF practices of mothers by using a pre-tested questionnaire. Eight local health extension workers (i.e., at out of their kebele to prevent bias) collected the data, and two nurses, including the principal investigators, served as supervisors. Both the interviewers and supervisors were trained for one day on the objective of the research, data collection procedures, and interviewing approach. The WHO definition of EBF was adopted, which stated that nothing other than breast milk had been administered in the previous 24 hours prior to the interview. EBF was calculated by asking mothers of infants aged 0 to 6 months to provide information about their infant’s feeding history for the previous 24 hours.

### Study variables

The dependent variable was exclusive breastfeeding practice. The independent variables were:

- **Socio-demographic/economic** variables such as religion, ethnicity, responsible care givers of the child, age of mother, marital status, residence, occupation, maternal education, monthly income, husband education, husband occupation, and family size
- **Child characteristics** such as age, sex, birth order, birth type
- **Health service related factors** such as antenatal care (ANC) attendance, staff advice on child feeding during ANC, postnatal care (PNC) use, place of delivery, mode of delivery
- **Child feeding practices** such as colostrum feeding, pre-lacteal feeding, and early initiation of breastfeeding

The following operational definitions were applied, consistent with WHO definitions:

**Exclusive breastfeeding**. If an infant less than six months old took only breast milk and no additional food, water, or other liquids (with the exception of medicine and vitamins, if needed) during the 24-hours before the interview was conducted,
**Pre-lacteal feeding**. If an infant during the first three days of life takes something other than breast milk,
**Children ever breastfed**. If children born in the last 6 months during the data collection were ever breastfed, and
**Early initiation of breastfeeding**. If children born in the last 6 months were put to the breast within 1 hour of birth.

### Data processing and analysis

The data were coded and entered into the statistical software EPI Info version 3.5.4, then exported to SPSS version 20 for analysis. To describe respondents using various characteristics of interest and establish the prevalence of exclusive breastfeeding, descriptive statistics were utilized. The prevalence of EBF was determined as the ratio of infants under the age of 6 months who only fed breast milk in the 24 hours leading up to the survey to the total number of children in the same age group. Bivariable and multivariable logistic regressions were performed to examine the association between independent variables and EBF. In the multiple logistic regression models, odds ratios with 95% confidence intervals were calculated, and variables with p-values less than 0.05 were considered significantly associated with the dependent variable.

### Ethical consideration

The study was first approved by Bahir Dar Institute of Technology, faculty of Chemical and Food Engineering. Officials at various levels in the study area were notified via letters from Bahir Dar Institute of Technology, and an official letter of cooperation was obtained from the Horro district Health bureau. Before the interview, each respondent gave their informed verbal consent after explaining the study objectives for the participants. Participation was voluntary and mothers/caregivers signed (or provided a thumbprint if illiterate) a statement of an informed consent, after which they were interviewed. Respondents were given the option of not responding to any questions they didn’t want to answer, as well as assurances concerning the confidentiality of the data. Following the interview, the importance of EBF was discussed with those who had not done so previously, and they were encouraged to do so.

## Results

### Sociodemographic characteristics of the participants

Out of 649 mother-infant pairs sampled, 638 of them participated in the study making the response rate of 98.31%. The age of respondents ranged from 18 to 42 years old. The mean age was 30.08 years with standard deviation (SD) 4.69 years. Half (50.8%) of the study participants were in the age range of 20 to 29 years. More than a half 331 (51.9%) of the respondents were Protestant Christian followed by 176 (27.6%) Orthodox Christian; 90.1% were rural residents; and 508 (79.6%) are farmers by occupation. The majority 607 (95.1%) were Oromo, followed by 30 (4.7%) of Amhara ethnic group (Table 1). One hundred and seventy-eight (27.9%) of mothers had no formal education, while 267 (41.8%) had a grade 1–8 educational level. Almost all of the mothers, 615 (96.4%), are legally married. About sixty percent of the participants have average monthly household income of <1000 Ethiopian Birr, and 358(56.1%) of husband’s educational status were Primary (Grade 1–8). Six hundred and thirty-three (99.2%) mothers were the primary caregivers for their children; 361 (56.6%) gave birth to a male infant, and 621 (97.3%) having had a singleton birth (Table 1).

**Table 1:** Socio-demographic characteristics of study participants (n = 638).

Variable Name	Category	Number	Percent
Responsible care giver	Mother	633	99.2
	Grand mother	5	0.8
Religion of mother	Protestant	331	51.9
	Orthodox Christian	176	27.6
	Waqefata	88	13.8
	Muslim	43	6.7
Ethnicity	Oromo	607	95.1
	Amhara	30	4.7
	Gurage	1	0.2
Age of mother (years)	≤19	10	1.6
	20–29	324	50.8
	> = 30	304	47.6
Residence	Rural	575	90.1
	Urban	63	9.9
Marital status of mother	Married	615	96.4
	Separated	12	1.9
	Single	11	1.7
Mother’s educational status	No formal education	178	27.9
	Primary (Grade 1–8)	267	41.8
	Secondary (Grade 9–12)	160	25.1
	College and above	33	5.2
Occupation of the mother	Farmer	508	79.6
	House wife	62	9.7
	Merchant	31	9.7
	Employed	23	3.6
	Daily labourer	8	1.3
	Students	6	0.9
Households’ family number	≤4	267	41.8
	>4	371	58.2
Sex the of the child	Male	361	56.6
	Female	277	43.4
Infant age (month)	< 2	68	10.6
	2–3	280	43.9
	4–6	290	45.5
Type of pregnancy	Singleton	621	97.3
	Twin	17	2.7
Husband’s educational status	No formal education	62	9.7
	Grade 1–8	358	56.1
	Grade 9–12	163	25.5
	College and above	44	6.9
Average monthly income of household	≤1000	379	59.4
	>1000	259	40.6

### Obstetric and health care related factors of the participants

The majority of mothers in the study, 546 (85.6%), received at least one ANC visit, and 295 (46.2%) said they were counselled on breastfeeding, including EBF for the first six months. Sixty percent of them gave birth to their current child at a health facility, and 623 (97.6%) gave birth via spontaneous vaginal birth. Three hundred and sixty-six (61%) of the mothers attended PNC. About one third (34.5%) of the respondents have gotten information on breastfeeding from a health professional, 173 (27.1%) from a health development army, 92 (14.4%) both from a health professional and a health development army, 83 (13%) from a peer group, and 47 (7.4%) from the mass media. Sixty-five percent of the mothers had had two to four children (Table 2).

**Table 2:** Obstetric history and health service distribution of study participants (n = 638).

Variable Name	Category (n = 638)	Number	Percent
**Parity**	≤1	129	20.2
	2–4	415	65
	>4	94	14.7
Antenatal care attendance	Yes	546	85.6
	No	92	14.4
Number of ANC visit	≤1	115	18
	2–3	394	61.8
	>3	129	20.2
Breastfeeding counselling given during ANC visit	Yes	295	46.2
	No	343	53.8
Place of birth	Health Institution	381	59.7
	Home	257	40.3
Mode of birth	Normal/Vaginal	623	97.6
	Caesarean section	15	2.4
Postnatal care	Yes	389	61
	No	249	39
Source of EBF information	Health professional	220	34.5
	Mass media	47	7.4
	Peer-group	83	13
	HDA	173	27.1
	H/P &MM	23	3.6
	H/P &HDA	92	14.4

HDA-health developmental army, H/P-health professional, MM-mass media.

### Breastfeeding practices

Sixty-two percent of the mothers initiated breastfeeding within one hour of birth, and 32.8% of them initiated breastfeeding within 1–24 hours. The majority of the mothers (92%) had fed colostrum, and 51 (8%) of them had squeezed it out and discarded it. The main reasons for squeezing out colostrum were that they believed it might cause ill health effects on their infants 15 (2.35%), that it might not be digestible for the infants 25 (3.92%), and that it might block milk secretion 11 (1.72%) (Table 3).

**Table 3:** Distribution of mothers by their exclusive breastfeeding experiences (n = 638).

Variables	Category (n = 638)	Number	Percent
Breastfeeding initiation	Within one hour	394	61.8
	1–24 hour	209	32.8
	Not sure	35	5.5
Pre-lacteal feeding	Yes	14	2.2
	No	624	97.8
What Pre-lacteal	Butter	4	0.6
	Aabish/ fenugreek solution	5	0.78
	Cow Milk	2	0.3
	Water	3	0.5
Colostrum discard	Yes	51	8
	No	587	92
Reason of colostrum	It might cause ill health	15	2.35
	It might not be digestible	25	3.92
	It might block milk secretion	11	1.72
Current breastfeeding	Yes	638	100
	No	0	0
Breastfeeding frequency	≤8	24	3.8
	>8	614	96.2
Exclusively breast feed	Yes	449	70.4
	No	189	29.6
Additional food given for the child	Axmiet	30	4.7
	Cow Milk	77	12.1
	Formula Milk	25	3.9
	Tea/sugar solution	17	2.7
	water	40	6.3
Reason for giving additional food (drink)	Mothers left home for work	23	3.61
	Breast milk is not sufficient, and to make the child strong	70	10.97
	The baby open mouth, and she/he might get thirsty	68	10.66
	Mothers didn’t have enough milk	28	4.39

**Fig 1:**
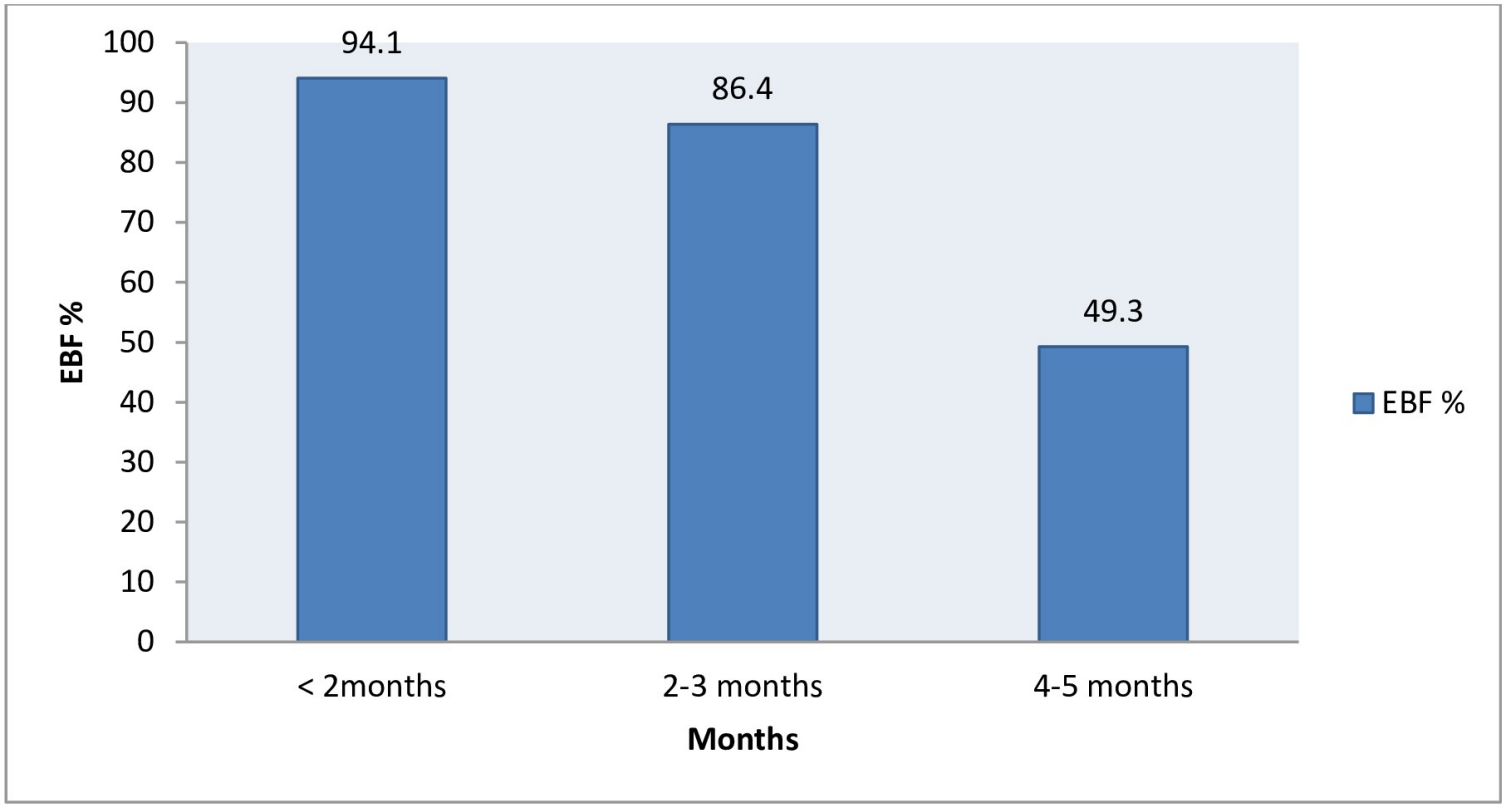
A month-specific lifetime exclusive breastfeeding among mothers.

Fourteen mothers (2.2%) gave pre-lacteal feed such as butter 4 (0.6%), aabish/fenugreek solution 5 (0.78%), cow milk 2 (0.3%), or water 3 (0.5%). Six hundred fourteen (96.2%) of the mothers were breastfeeding more than eight times per day. All respondents had ever breastfed their infant and were still doing so at the time of the survey. The proportion of EBF for infants aged less than six months in the study area was 449 (70.4%) as measured by the last 24-hour recall period preceding the survey date. The proportion of infants who are exclusively breastfed declined from 94.1% of infants aged <2 months to 86.4% of those aged 2 to 3 months, and further to 49.3% among infants aged 4 to 5 months, as depicted in Fig 1 **below**.

The non-exclusively breastfeeding mothers supplemented their breast milk with some addition of foods like cow milk 77 (12.1%), Axmiet (cereal-based fluids) 30 (4.7%), water 40 (6.3%), formula milk 25 (3.9%), and tea/sugar solution 17 (2.7%). The common reasons reported for not feeding breast milk only during the first six months of life include the following: the mothers had left home for work; perceiving that breastfeeding only is not sufficient for an infant after 4 or 5-months old; to make the child more stronger; the infant was challenging to eat (drink) (i.e., the baby opened its mouth when the mother took food); perceiving that the baby might get thirsty; and the mother did not have adequate milk (Table 3).

### Determinants of exclusive breastfeeding

The bivariable analysis showed that factors such as responsible care givers for the baby, marital status, maternal education and occupation, parity, number of ANC follow-up, hearing BF information during ANC, having had PNC follow-up, place of birth, infant age, mode of birth, average monthly income, colostrum discarding, and initiation of BF time were significantly associated with EBF.

Receiving BF information during ANC follow-up, PNC follow-up, infant age, mode of birth, total monthly income, and initiation of BF time were all independent factors associated with EBF practice in the multivariable logistic regression analysis. Mothers who got BF counselling during ANC were four times higher than their counterparts to exclusively breastfeed their babies (AOR = 4.15, 95% CI (2.36, 7.30)). Mothers who attended the PNC follow-up were five times higher than those who did not to exclusively breastfeed their children (AOR = 4.74, 95% CI (2.92, 7.70)) (Table 4).

**Table 4:** Factors associated with EBF practice of mothers among study participants (n = 638).

Variable Name	Category	EBF Practice		COR (95% CL)	AOR (95%CL)
		No	Yes		
Responsible child’s care giver	Mother	185(29.2)	448(70.8)	9.69 (1.08, 87.25) *	0.68 (0.03, 17.83)
	Grand-Mother	4(80.0)	1(20.0)	1	1
Mother’s age (years)	15–19	6(60.0)	4(40.0)	0.31(0.09, 1.12)	0.40(0.05, 3.15)
	20–29	87(26.9)	237(73.1)	1.26(0.89, 1.78)	0.78(0.41, 1.49)
	≥30	96(31.6)	208(68.4)	1	1
Residence	Rural	116(28.9)	409(71.1)	1.42(0.82, 2.44)	1.93(0.74, 5.08)
	Urban	23(36.5)	40(63.5)	1	1
Mothers marital status	Married	173(28.1)	442(71.9)	4.47(1.29, 15.47) *	5.20(0.63, 43.17)
	Separated	9(75.0)	3(25.0)	0.58(0.10, 3.51)	1.35(0.08, 21.79)
	Single	7(63.6)	4(36.4)	1	1
Mothers educational status	No formal education	66(37.1)	112(62.9)	1	1
	Grade 1–8	68(25.5)	199 (74.5)	1.73(1.14–2.60) *	1.41(0.76–2.69)
	Grade9-12	38(23.8)	122(76.2)	1.89(1.18–3.04) *	0.67(0.28–1.59)
	College and above	17(51.5)	16(48.5)	0.56(0.26–1.17)	0.28(0.06–1.26)
Mothers Occupation	Employed	13(56.5)	10(43.5)	1	1
	Farmer	141(27.8)	367(72.2)	3.38(1.45, 7.89) *	1.86(0.33, 10.44)
	House Wife	20(32.3)	42(67.7)	2.73(1.02, 7.28) *	2.10(0.43, 10.23)
	Merchant	8(25.8)	23(74.2)	3.74(1.18, 11.83) *	3.93(0.61, 25.16)
	Daily Labourers	3(37.5)	5(62.5)	2.17(0.42, 11.30)	2.01 (0.13, 32.23)
	Students	4(66.7)	2(33.3)	0.65 (4.29, 0.10)	0.46 (0.03, 6.93)
HH family members	≤4	69(25.8)	198(74.2)	1.37 (0.97, 1.95)	1.19 (0.61, 2.32)
	>4	120(32.3)	251(67.7)	1	1
Parity	≤1	37(28.7)	92(71.3)	1.76(1.01, 3.09) *	1.59 (0.50, 5.02)
	2–4	113(27.2)	302(72.8)	1.90(1.19, 3.01) *	0.96(0.46, 2.01)
	>4	39(41.5)	55(58.5)	1	1
Number of ANC Visit	≤1	67(58.3)	48(41.7)	0.23(0.13, 0.39) *	0.49(0.19, 1.22)
	2–3	91(23.1)	303(76.9)	1.05(0.66, 1.68)	0.87(0.43, 1.77)
	>3	31(24.0)	98(76.0)	1	1
BF information on ANC	Yes	44(14.9)	251(85.1)	4.18(2.84, 6.14) *	**4.15(2.36, 7.31)** *
	No	145(42.3)	198(57.7)	1	1
PNC follow up	Yes	61(15.7)	328(84.3)	5.69(3.93, 8.23) *	**4.74(2.92, 7.70)** *
	No	128(51.4)	121(48.6)	1	1
Place Of delivery	Health Institute	93(24.4)	288(75.6)	1.85(1.31, 2.61) *	0.78(0.44, 1.37)
	Home	96(37.4)	161(62.6)	1	1
Infant age in month	0–1	4(5.9)	64(94.1)	16.45(5.84, 46.35) *	**12.14(3.83, 38.46)** *
	2–3	38(13.6)	242(86.4)	6.56(4.34, 9.89) *	**8.62(5.00, 14.85)** *
	4–6	147(50.7)	143(49.3)	1	1
Birth type	Single	178(28.7)	443(71.3)	4.56(1.66, 12.53) *	**8.91(3.86, 43.21)** *
	Twin	11(64.7)	6(35.3)	1	1
HH monthly income	≤1000	99(26.1)	280(73.9)	1.51(1.07, 2.12) *	**1.96(1.16, 3.32)** *
	>1000	90(34.7)	169(65.3)	1	1
Colostrum discard	Yes	30(58.8)	21(41.2)	0.26(0.15, 0.47) *	0.85(0.37, 1.91)
	NO	159(27.1)	428(72.9)	1	1
Initiation of BF time	1–24 hour	90(43.1)	119(56.9)	1	1
	Immediately (<1hour)	92(23.4)	302(76.6)	2.48(1.73, 3.56) *	**1.94(1.13, 3.35)** *
	Not sure	7(20.0)	28(80.0)	3.03(1.26, 7.24) *	2.80(0.92, 8.48)

HH- household,

*P-value <0.05 (Significant).

The odds of exclusively breastfeeding are twelve times and nine times higher among mothers with infants aged less than a month and 2 to 3 months as compared to mothers with 4 to 6-month-old infants (AOR = 12.14, 95% CI (3.83, 38.46)) and (AOR = 8.62, 95% CI (5.00, 14.85)) respectively. Similarly, mothers who had a singleton birth were nine times higher to exclusively breastfeed than mothers who had a twin birth (AOR = 8.91, 95% CI (3.86, 43.21)). Mothers from households with a monthly income of ≤1000 Ethiopian Birrs were two times higher to exclusively breastfeed than mothers from households with a monthly income of >1000 Ethiopian Birrs (AOR = 1.96, 95% CI) (1.16, 3.33). This study also revealed that mothers who initiated BF within one hour of birth were two times more likely to exclusively breast feed as compared with those who did not (AOR = 1.94, 95% CI (1.13, 3.35)) (Table 4).

## Discussion

This study assessed breastfeeding practices and determinants of EBF in the Horro district, Ethiopia. Infants who were ever breastfed, breastfed in the first hour, fed something other than breast milk before breast milk was regularly supplied (also known as pre-lacteal feeding), and exclusively breastfed were all included in the study.

The majority of mothers (61.8%) initiated BF within one hour of the baby’s birth, which is good according to WHO indicator [21]. This is comparable with the findings from Jimma Arjo, which was (62.6%) [22]. However, it was relatively higher when compared with a review study of the Middle East which was (34.3%) [23]. This study’s findings were lower than those of previous studies in Ambo town (71.2%) and EDHS 2016 (73%) Ethiopia [14, 24]. This could be attributed to the harmful effects of cultural and customary malpractices on BF habits, as well as a lack of understanding about the need to initiate BF within one hour. Among the study participants, 51 (8%) of them had squeezed and discarded the colostrum with the assumption that it might cause some illness 15 (2.35%), it might not be digestible 25 (3.92%) or it might block milk secretion 11 (1.72%). This finding is lower than the findings of Goba wereda, South East Ethiopia, where 35% of mothers discarded colostrum due to the cultural belief that it could cause abdominal cramps in the infant [25]. These assumptions should be corrected through appropriate counselling during ANC fellow-up.

In this study, a pre-lacteal feeding such as butter 4 (0.6%), fenugreek 5 (0.78%), cow milk 2 (0.3%), and water 3 (0.5%) was given for the infants with the assumption of softening the child’s stomach and making the overall proportion of pre-lacteal feeding 14 (2.2%). This proportion is lower than the national pre-lacteal feeding (8%) reported by EDHS 2016 [14], as well as the findings of other studies like Sierra Leone (19.2%), Bahir Dar city (26.3%), and Goba wereda of Ethiopia (17.2%) [19, 25, 26]. These differences might possibly be due to the socio-cultural and study time differences between the study areas. Designing and implementing effective behavioral change communications is highly needed to improve the situation.

According to the study, all mothers had ever practiced BF, which is comparable with the national ever BF practice (97%) [14]. The study revealed that the proportion of EBF was 70.4%. This finding is good according to the WHO indicator [21], but it is greater than the global nutrition target of meeting 50% of EBF by the end of 2025 [9]. This finding was similar to reports from studies conducted at Goba (71.3%), and Enderta, Ethiopia (70.02%) [25, 27]. The findings of this study were greater than the EDHS 2016 report (58%) and Bedele town (43.6%) [14, 17], and other countries’ findings such as Nairobi (45.5%), Abu Dhabi (44.3%), Angola (51.5%), Lagos Nigeria (3.6%) and Bhubaneswar India (21.2%) [20, 28–31]. This study’s finding is lower than those of a previous study conducted in Ambo wereda, Ethiopia (82.2%) [24]. All this variation might be due to methodological, sample size, study time, sociodemographic, economic, and cultural differences across areas. From this study, 189 (29.6%) of mothers did not practice EBF due to the assumption that mothers didn’t have enough breast milk 28 (4.39%), mothers left home for work 23 (3.61%), breast milk is not sufficient and to make the infant stronger 70 (10.97%), and the infant opens their mouth when they eat and might get thirsty 68 (10.66%). As supported by some studies, this practice is common in many developing countries [22, 32]. The month-specific prevalence of EBF decreases progressively with infant age, with a prevalence of 94.1%, 86.4%, and 49.3% at 2 months, 2–3 months, and 4–5 months, respectively. This finding indicates that the older a child is, the more likely that he/she is non-exclusively breastfed, which is similar to the conclusion drawn at the national level, Jimma town, Jimma Arjo, and Sorro district of Ethiopia [14, 18, 22, 33].

The factors which affect EBF duration are not only many and complex, but they work differently in different situations. According to this study, BF information (counselling) given on ANC, PNC follow-up, infant age, birth type of infant, total monthly income, and initiation of BF time are the variables that were significantly associated with EBF practice during the multivariable analysis. Women who had gotten BF information during ANC follow-up (not the number of ANC) during their pregnancy period were four times higher to practice EBF compared to those who did not have the information. This is consistent with the study findings from Debre Markos and Addis Abeba in Ethiopia [34, 35], and other countries such as Egypt and Pakistan [36, 37]. This could imply that those mothers who were counselled and well informed about BF practiced EBF well. The Baby Friendly Hospital initiative highlights that ANC information is one of the key clinical practices in protecting, promoting, and supporting breastfeeding. Global recommendations on EBF for the first 6 months, the risks of giving formula or other breast-milk substitutes, and the fact that BF continues to be important after 6 months when other foods are given, is one of the minimum protocols for antenatal discussion of BF [38].

The odds of EBF were 4.75 times higher among mothers who received PNC as compared to those who did not receive the care. Similar findings have been reported in Addis Abeba, Enderta Wereda, Debre Markos, and Awi zones of Northern Ethiopia, as well as Lagos, Nigeria [27, 30, 34, 35, 39]. According to EDHS 2016, in Ethiopia, 81% of women do not receive PNC follow-up, and the low uptake could impact EBF practice [14]. In this study, parity was found to be a significant variable during the bivariable logistic regression analysis but not during the multiple logistic regression analysis. However, studies conducted in Gwanda District, Zimbabwe and North Jordan [13, 40], concluded that multi-parity was a major predictor of exclusive breastfeeding. These variations might be due to the cross-cultural preferences and sociodemographic characteristics differences across a region. Another possible reason for this difference might be that in this study, primiparas mothers might be the one who had received breastfeeding counselling during antenatal care, and by any other chances.

In this study, EBF decreased as the age of the infant increased. Infants at younger age (≤ 1 month) were 12 times and those who were at the age of 2–3 months were 8.62 times higher to be exclusively breastfed as compared to infants above 4 months. This finding was consistent with a conclusion drawn by study findings such as Goba district, Jimma town, Jimma Arjo, and Enderta wereda in Ethiopia [18, 22, 25, 27] and other countries such as Nigeria and West African State where EBF decreases by 33% as the infant age (in months) increases [41, 42]. This is due to the mother’s thinking that breast milk alone was not enough as the infant got older and older, which is common in many developing countries [22, 32]. Hence, they provide additional food items other than breast milk. Moreover, it might be due to the fact that post-partum care is traditionally given in the first few months when mothers are confined at home [43]. It can also be attributed to the fact that short birth intervals/spacing, as in the case of developing countries including Ethiopia, reduces the duration of EBF of infants in the first six months.

Mothers with singleton births were 8.91 times higher to exclusively breastfeed as compared with mothers who gave birth to twin babies. This finding is in agreement with studies conducted on Ghanaian twins that reported twins and other higher-order multiple births are less likely to be EBF at six months compared to their singleton counterparts [44]. This could be possibly due to some mothers’ worry that they won’t make enough milk for their twins and a lack of correct information and support from their family. But if they feed their infants on demand when they are hungry, their breasts can actually produce up to double the amount of milk their babies need. The law of supply and demand applies to nursing mothers of twins and multiples. If a mother breast feeds when her babies want to eat, they can trust their body to supply enough milk. A low milk supply can almost always be corrected by nursing more often. If their babies aren’t emptying their breasts, they may need to pump [8]. Moreover, it might be due to a lack of enough support from the husband or other family members, which makes caring and EBF a difficult scenario [45, 46] for twin birth mothers.

Households’ monthly income was statistically significantly associated with EBF practice. Mothers from households with an average monthly income of ≤ 1000 Ethiopian Birrs were two times higher to exclusively breastfeed than those whose income was >100 Ethiopian Birr. There are contradictory findings regarding the relationship between income and EBF. In another study, there was no relation between EBF and the income of the mother [47]. The findings of this study were in contradiction with some findings obtained from studies in Northwest Ethiopia, Nigeria and West Africa [41, 42, 48]. This study indicates that when the mother earned a high income, the rate of EBF decreased, which is consistent with studies done in East Gojjam and Addis Ababa, Ethiopia [35, 46]. Similarly, improved socioeconomic status both at individual and societal levels was found to be a negative factor for EBF in a multilevel analysis of factors associated with non-EBF in five East and Southeast Asian countries [49]. This conclusion could be explained by the fact that women with greater incomes are less likely to stay at home throughout the day, which could compromise the practice of exclusive breastfeeding. Another reason could be the cost of infant formula and cow’s milk, which are prohibitively expensive unless the family has a greater income.

This study revealed that mothers who initiated BF within one hour after birth were two times higher to exclusively breastfeed than those who initiated BF after one hour of birth. The finding was in agreement with a study conducted in Southern Ethiopia, which concluded that those women who breastfed their babies within one hour had a higher rate of EBF till six months after birth [33]. Results from a population-based Study of Colorado mothers also showed that initiating BF in the first hour after birth resulted in a longer BF duration, which might increase the chances of EBF as well [50]. From the control and experimental study in PGIMER, Chandigarh Institute, the early-initiated group fed for an average of 161 days, or almost 40% longer than the other group’s 96-day average [51]. This could be due to a positive effect of early initiation of BF both on the likelihood of EBF and the overall duration of breastfeeding [51, 52].

## Conclusion

In the study area, all mothers have ever breastfed even if there are some sub-optimal BF practices such as delayed initiation of BF (38.2%), colostrum discarding (8%), and pre-lacteal feedings (2.2%), which may affect the health of both the mother and the infant. The study revealed that the proportion of EBF using the 24-hour recall method was 70.4%, which is good but lower than the WHO recommendation (> 90%). The practice of EBF in the study area meets the global nutrition target of 2025 of meeting at least 50% EBF. From the study findings, breastfeeding information on ANC follow-up, PNC follow-up, infant age, type of pregnancy (singleton or multiple), average monthly income, and early initiation of BF were factors associated with EBF practice. Counselling of pregnant women about BF issues during ANC services, enabling all mothers to get access to PNC services, and encouraging BF initiation within one hour were recommended in order to increase the proportion of EBF practices in the study area.

## Supporting information

S1 Appendix (DOCX)
