# Supplementary material for: Breastfeeding practice and factors associated with exclusive breastfeeding among mothers in Horro District, Ethiopia: A community-based cross-sectional study
Source: PLoS One. 2022 Apr 27;17(4):e0267269. doi: 10.1371/journal.pone.0267269 (PMC9045649; doi:10.1371/journal.pone.0267269)
Supplement: S1 Appendix — (DOCX) [file pone.0267269.s001.docx]

# APPENDIX I

**BAHIR DAR UNIVERSITY**

**FACULTY OF CHEMICAL AND FOOD ENGINEERING,**

**Applied Human Nutrition Department**

# English Version Informed Consent Form

**Title:** Breastfeeding practice and factors associated with exclusive breastfeeding among mothers of children less than 6 months of age in Horro District, Oromia region, Ethiopia: a community-based cross-sectional study

Principal Investigator: **Debela Daba Jebena**

Address: Bahir Dar Institute of Technology, Department of Applied Human Nutrition

**General Information about Research**

You and your child are being invited to participate in a study that seeks to determine breastfeeding practice and the various factors that influence the exclusive breastfeeding patterns of mothers. This is important to know because the information from this study could be used to better advice breastfeeding mothers.

If you agree to take part in this study, you will be asked some personal questions such as your age, level of education, where you live, the number of your family size; your baby’s birth information, such as birth type of birth, birth order, age; your biomedical history such as delivery type, antenatal care, postnatal care, and breastfeeding advice received. This would take about 40-50 minutes.

**Possible Risks and Discomforts**

There would be no hurt to you and your baby. However, the time to be spent answering the questions may pose some inconvenience to you. It is possible that some of the questions to be asked may pose some discomfort or intrude on your privacy. You are free to choose not to answer any question(s) that you are not comfortable with or do not wish to discuss and you may stop the interview at any time.

.

**Responsible Person of the study:**

- If you have any questions about the survey I am ready to respond.
- Do you have any questions about the survey? Please let me know if anything I have stated is not clear and I will be happy to explain it further to ensure you understand.

Signature of the interviewer certifying that informed consent has been given verbally by respondent.

*_____________________________ ___________ __________*

Signature of a person who take the consent Date Time

*________________________________*

Name of a person who take the consent

Are you willing to participate in the study?

Yes_______ No__________

***_____________________________ ___________ __________***

Witness Signature (if only the participant unable to write) Date Time

*______________________*

Interviewee Code

# APPENDIX II

**BAHIR DAR INSTITUTE OF TECHNOLOGY**

**DEPARTMENT OF APPLIED HUMAN NUTRITION**

**Prevalence and determinants of exclusive breastfeeding among mothers having children less than six months in Horro district, Oromia region, Western Ethiopia.**

## English version study questionnaire

**Survey General Information**

| No | Questions and Explanations | Alternatives/Answers |
| --- | --- | --- |
| 01 | Questionnaire Number consist of the Region and Woreda code, Kebele & Household Number  **(***To be filled before interview)* | Region _**Oromia**  Kebele Name _______  Woreda __**Horro**  House Number _________ |
| 02 | Interviewer and Field Supervisor Name & Signature.  Interviewer:  Field supervisor : | Name፡ ________________  Signature፡ ________  Name፡ ________________  Signature፡ ________ |
| 03 | Number of days the household visited for interview | One (01)  Two (02)  Three (03) |
| 04 | Date the questionnaire started and completed  Date Interview Started:  Date interview Completed: | [_____\|_____\|_____\|  dd MM YY  [_____\|_____\|_____\|  dd MM YY |
| 05 | Questionnaire Status | Completed (01)  Absent at Home (02)  Unwilling to Participate (03)  Other (Specify) (04)_____________ |

| **PART ONE: SOCIO-DEMOGRAPHIC VARIABLES** | | | |
| --- | --- | --- | --- |
| **NO** | **Question and Explanations** | **Response/ Alternatives** | **Skip to/Remark** |
| 01 | Who is the main responsible caregiver for child? | - Mother (01) - Grand-mother (02) - Sister (03) - Servant (04) |  |
| 02 | What is your religion? | - Orthodox Christian (01) - Protestant (02) - Wakefata (03) - Muslim (04) - Non-believer (05) - Other (specify) ____________ (06) |  |
| 03 | What is your ethnicity? | - Oromo(01) - Amhara(02) - Gurage (03) - Others………..…………….. (04) |  |
| 04 | What is you residence? | - Urban(01) - Rural(02) |  |
| 05 | How old are you?  *(Age in completed years)* | ……………………. |  |
| 06 | What is your current marital status? | - Married and living together (01) - Single (02) - Widowed (03) - Divorced (04) - Separated 05 |  |
| 07 | What is your educational status? | - No formal education (01) - Grade1 – 8 (02) - Grade 9 – 12 (03) - College and above (04) |  |
| 08 | What is your occupation? | - Farmer/Agricultural worker (01) - Daily labourer (02) - merchant (03) - employed (04) - House wife (05) - Other (specify) ____________ (06) |  |
| 09 | How many Family sizes in Home? | ……………….. |  |
| 10 | Parity | ……………… |  |
| 11 | What is the sex of child? | - Male(01) - Female(02) |  |
| 12 | The age of your infant(in months) currently is | ………………….. |  |
| 13 | Type of birth | - Single (01) - Multiple/Twin/(02) |  |
| 14 | What is the highest educational status of your Husband? | - No formal education (01) - Grade 1 – 8 (02) - Grade 9 – 12 (03) - Diploma and above (04) |  |
| 15 | What is the means of your family income (Only for the purpose of estimating their income) | - Selling cattle, Goat, sheep and etc (01) - Selling (renting) land (02) - Selling different household materials (03) - Selling grains(04) - Working as daily labourer (05) - Shopping (06) - Monthly Salary (07) - Others………………………(08) |  |
| 16 | Depending on Question number 18, how much is your monthly income? | ………………….. |  |
| **Part II: Maternal and child Health service related factors** | | | |
| 17 | Did you visit health facility for ANC during your pregnancy for this child? | - Yes (01) - No (02 | If No, skip to Q20 |
| 18 | If yes to Question No 20, how many times did you receive (number of antenatal care) during your time of Pregnancy for this child? | …………………….. |  |
| 19 | Have you ever been given information/counselling on BF at ANC visit? | - Yes (01) - No (02) |  |
| 20 | Where did you gave birth to  this child /Place of delivery/ | - Home (01) - Health Institution (02) |  |
| 21 | Type of delivery | - Normal/Vaginal (01) - Caesarean section (02) |  |
| 22 | Have you received post-natal care (PNC)? | - Yes (01) - No (02) |  |
| 23 | What is your source of nutrition information about Exclusive Breast feeding? | - Health professional (01) - Mass media (02) - Per-group (03) - Health development arm (04) |  |
| 24 | If you answer is mass media, which one? | - Radio (01) - Television (02) - Newspaper (03) - Internet access (04) |  |
| **Knowledge and Feeding Practice based questionnaire** | | | |
| 25 | How long after birth did you first out the child to breast feed? | - Immediately (01) - ____Hours (If less than 24 hrs record) (02) - _____Days (03) - Don't know/not sure/ (04) |  |
| 26 | Did you give the child (NAME) pre-lactation food/fluid? | - Yes (01) - No (02) | If no, skip to Q 28 |
| 27 | If yes, what did you gave him (her)? | - Water (01) - Butter (02) - Milk (03) - other (Specify) (04) |  |
| 28 | Did you squeeze out and throw the first milk (colostrum)? | - Yes (01) - No (02) | If No, skip to Q 30. |
| 29 | IF yes, Why? | ………………………. |  |
| 30 | Are you still breastfeeding? | - Yes (01) - No (02) | If yes, skip to Q32 |
| 31 | If you say no for Q 33, why? | …………………. |  |
| 32 | How many times in the last 24 hours you breastfed? | _____________times. |  |
| 33 | Did you give the child additional food or fluid other than breast milk in the past 24 hours? | - Yes (01) - No (02) | If No, Skip Q34 and 35 |
| 34 | If yes, what ingredients you gave? (More than one answer is possible) | - Cow’s milk (01) - Butter (02) - Tea, Sugar solution (03) - Formula milk (04) - Axmiet/Bula (05) - Other (specify) (06) |  |
| 35 | Why you are interested to give additional food for the child? | ----------------- |  |

#

# APPENDIX III

**Dhaabbata Techonolojii Bahir Daritti**

**kutaa barumsaa” Chemical and Food Engineering”**

sadarkaa sirna Harma qofaa hoosisuu Haadholii Daa’imman Umrii ji’a Jahaa gadi tahan qabanii fi dhimoota murtessa issa ta’aan sadarkaa mana manatti funaanuuf, aanaa Horroo, Naannoo Oromiyaa, dhiha Itoophiyaa.

# Afaan Oromo Version Informed Consent Form

**Waliigaltee**

**Title:** Breastfeeding practice and factors associated with exclusive breastfeeding among mothers of children less than 6 months of age in Horro District, Oromia region, Ethiopia: a community-based cross-sectional study

**Mata duree:** sadarkaa sirna Harma qofaa hoosisuu Haadholii Daa’imman Umrii ji’a Jahaa gadi tahan qabanii fi dhimoota murtessa isaa ta’an sadarkaa manaa manatti funaanuuf, aanaa Horroo, Naannoo Oromiyaa, Itoophiyaa.

Hogganaa Qorannichaa: **Dabalaa Dhaabaa**

Teessoo: Dhaabbata Techonolojii Bahir Daritti, Muummee barnootaa Applied Human Nutrition

**Odeeffannoo Walii gala Waa’ee Qorannichaa**

Ati fi da’imnikee qorannoo waa’ee harma qofaa hoosisuu fi dhimmoota muteessaa isaa adda baasuu jedhu irratti akka hirmaattaniif afferamtaniittu. Qorannoon kun baay’ee barbaachisaa fi haadhoolii harma hoosisan gorsuuf gargaara.

Qorannoo kana irratti hirmaachuuf fedha yoo qabaattaniif dhimmoota dhuunfaa keessanii kan akka Umurii, sadarkaa barnootaa, bakka jireenyaa, baay’ina miseensa maatii keessanii fi akkasumas dhimmootaa waa’ee Daa’ima keessanii gosa dhalootaa, Da’ima meeqaffa akka isniif ta’e, umurii; dabalataanis Gargaarsa fayyaa kan akka hordoffi yeroo ulfaa, Da’umsa boodaa fi gorsa waa’ee harma hoosisuu fudhachuu keessan isin gaafanna. Kanaafis yeroo keessan daqiiqaa 30- 45 isinitti fudhachuu danda’a.

**Rakkoolee isin Mudachuu Danda’an**

Isiniif fi mucaa keessan irratti mudhaan qaqabu hin jiru. Garuu gaaffi qorannoo kana deebisuuf yeroo keessan aarsaa gochuu isin gaafata. Akkasumas gaaffileen tokko tokko isinitti toluu dhiisuu danda’u. Isin garuu gaaffi isinitti hin tolle dhiisuu ykn gidduutti addaan kutuu fi kan feetan qofa deebisuuf mirgi keessan eegamaadha.

Hogganaa nama daataa funaanuu

Gaaffiin isiniif hin galle yoo jiraate ani isiniif deebisuuf qophaa’aa dha.

Gaaffii ifa isiniif hin taane qabduu? Na gaafadhaa

Mallattoo namni gaafatuu walii galtee isaa afaaniin mirkaneessu isaa.

Maqaa nama ragaa funaanee: ………………………………………

Mallattoo nama ragaa funaanee: ………………………………….

Guyyaa: …………………………………

Hirmaachuudhaaf fedhii qabduu? Eeyyee---------------- lakki/miti------------------------

___________________________________ _____________

Maqaa fi Mallattoo nama yeroo walii galteen kun raawwate argee Guyyaa

Lakk eenyummaa Qoranoof kenname: _______________

# APPENDIX IV

**Dhaabbata Techonolojii Bahir Daritti**

**Muummee Barnoota Applied Human Nutrition**

sadarkaa sirna Harma qofaa hoosisuu Haadholii Daa’imman Umrii ji’a Jahaa gadi tahan qabanii fi dhimoota murtessa issa ta’aan sadarkaa mana manatti funaanuuf, aanaa Horroo, Naannoo Oromiyaa, Itoophiyaa

Afaan Oromo version study questionnaire

Gaaffiillee qorannoo Afaan Oromootiin qophaa’e

| **Section 01: Funaansa odeeffanoo walii gala** | | |
| --- | --- | --- |
| **Lakk** | **Gaaffii fi Ibsa** | **Filannoo/Deebii** |
| **01** | Maqaa Naannoo,Aanaa, gandaa fi lakkoofsa manaa  **(***osoo gara gaafii fi deebii waa’ee qoranootti hin galiin dura bakka duwwaan fulduraa kana guutaa)* | Naannoo: **oromiyaa**  Aanaa: **Horroo**  Ganda…….………………..  Lakk. Manaa: ……… |
| **02** | Maqaa Namaa odeeffannoo funane/ nama gaafii fi deebii ummata waliin geggeesse  Maqaa ogeesa oddeefanoo funanee:    Hoganaa odeefanoo funanee : | Maqaa-------------------------------  Mallattoo፡ ________  MAqaa ________________  Mallattoo፡ ________ |
| **03** | **Baay’ina guyyaa Manichi Gaaffiidhaaf Hordofame** | **Guyyaa Tokko (01)**  **“ Lama (02)**  **“ sadii ( 03)** |
| **04** | Guyyaa odeeffanoo funanuu eegalee fi guyyaa itti xumuree  Guyyaa odeeffanoo funanuu eegalee      Guyyaa itti xumuree : | [_____\|_____\|_____\|  Guyyaa ji’aa bara  [_____\|_____\|_____\|  Guyyaa ji’aa bara |
| **05** | Haala Gaafiillee qoranoo  : | Xumuraniru(01)  Hin xumuree (02)  Hirmachuuf fedhii hin qaban (03)  Sababa biroon yoo jiraate sanduuqa keessatti guuti ( ___________________) (04) |

| **KUTAA TOKOFAA: GAFILEE HAWASSUMAA FI DINAGDEE** | | | |
| --- | --- | --- | --- |
| Lakk | **Gaaffii fi Ibsa** | Deebii | Adeemsa |
| 01 | Itti gafatamummaan daa’ima kan kununsuu eenyuudha? | - Haadha(01) - Akkoo/Haadha Abbaa(02) - Maatii(03) - Obbooleetii(04) - Hojeetuu(05) - Kan biroo ………………….(06) |  |
| 02 | Amantaan keessan maaliidha? | - Ortodoksii (01) - Protestantii (02) - Waaqefataa (03) - Musliima(04) - Amantaa hin qabu (05) - Kan biro(ibsi) ________________ (06 |  |
| 03 | Qomoon keessan maaliidha? | - Oromoo(01) - Amharaa(02) - Guragee(03) - Kan biiroo(ibsi)--------------------(04) |  |
| 04 | Bakki jireenyaa | - Baadiyaa(01) - Magaala(02) |  |
| 05 | Umriin keessan meeqadha?  *(Bakka duwwan guutaa)* | -------------------- |  |
| 06 | Haalli gaa’ela keessanii haala kamidha? | - Abbaa manaa waliin jirra(01) - Dhuunfaakooti/Hin heerumne (02) - Fooniin addunyaa kanarra boqotan(03) - Wal hiikne (04) - Bakka adda addaa jirra (05) |  |
| 07 | Sadarkaa barumsaa isiin irra geessan maaliidha? | - Barreessuus Dubbisuus hin danda’u(01) - Barreessuf dubbisuu qofa(02) - Kutaa 1-8 (03) - Kutaa 9-12 (04) - Diploomaa fi isaa ol(05) |  |
| 08 | Hojii kee/ haadha manaa/ maaliidha? | - Qonnaarratti kan bobateedha(01) - Hojii guyyaa ogummaa qabudha.(02) - Hojii guyyaa ogummaa hinqabnedha.(03) - Daldalaarratti kan bobaneedha(04) - Miindeffamtuu dha (05) - Haadha manaa qofa dha (06) - Kan biro-------------------------(07) |  |
| 09 | Miseensi maatii keessanii meeqa? | ---------------------- |  |
| 10 | Daa’imni kun, ijoollee keessaniif meeqaffadha? | --------- |  |
| 11 | Saalli Daa’ima keessanii maali | - Dhiira (01) - Dhalaa(02) |  |
| 12 | Umrii daa’ima kanaa meeqa? | ------------------ |  |
| 13 | Gosti dhaloota Daa’ima kanaa? | - Qofaa (Qeenxee) dhalatte/te(01) - Lakkuu dhalatan(02) |  |
| 14 | Sadarkaan barnoota abba manaakeetii | - Barnoota iddilee hin baranne (01) - Kutaa 1-8 (02) - Kutaa 9-12 (03) - Diploomaa fi isaa ol(04) |  |
| 15 | Maddi galii keessanii maali dha? | - Loon, Re’ee, Hoolaa fi k.k.f. gurguruudhaani(01) - Lafa gurguruu/ kirayeessuudhaani(02) - Meeshaalee garaagaraa kan mana keessaa gurguruutiin(03) - Midhaan gurguruudhaan(04) - Hojii humnaa hojjechuudhaan(05) - Suuqii/daldala(06) - Miindaa ji’aa(07) - Kan biro--------------------------(08) |  |
| 16 | Gaaffii 18^ffaa^  irratti hundaa’uun hammi galii keessanii ji’aan meeqa ta’a? | ---------------- |  |
| **Kutaa lammaffaa:Gaaffiilee Tajaajila fayyaa Haadhaa fi Mucaa waliin wal qabate.** | | | |
| 17 | Yeroo garaatti battu wajjira fayyaa adeemuun gorsa ogeesaa fudhachaa turtanii? | - Eeyyee (01) - Miti (02 | Miti yoo jette gara G-20ti darbi |
| 18 | Yoo eeyyeen jettan, yeroo meeqaaf mana yaalatti deddebi’uun gorsa fudhachaa turta? | --------- |  |
| 19 | Yeroo hordoffii Da’umsaa taasisaa turtetti Barnoonni/gorsi waa’ee harma hoosisuu siif kennameeraa? | - Eyyee(01) - Miti/Lakki (02) |  |
| 20 | Daa’ima ammaa kana eesatti deessan? | - Mana jireenya (01) - Mana yaalaa (02) - Kan biraa (Ibsi)__________(03) |  |
| 21 | Haalli da’umsa keessanii akkam ture? | - Nageenyumaan deesse(01) - Opireeshiniin deesse(02) |  |
| 22 | Erga deessanii booda Gorsa/ barumsa mana yaalaa irraa fudhatanituu? | - Eeyeen(01) - Miti (02) |  |
| 23 | Maddi odeeffannoo waa’ee Daa’ima hanga ji’a jahatti harma qofaa hoosisuu irraa argattan maali? | - Ogeesa fayyaa (01) - Kutaa odeefanoo danbalii (Radiyoo, televezhinii)(02) - Garee gitaarra/Hiriyyaa irraa(03) - Garee shanee sirna/humna guddistu fayyaa(04) |  |
| 24 | Yoo maddi odeefannoo kee karaa danbalii ykn dubbisaa ta’ee isaa keessa karaa kamiin odeeffannoo argata? | - Raadiyoo (01) - Televisizhinii (02) - Gaazexaa(03) - Interneetii (04) |  |
| **Gaaffilee Beekumsaa fi Ogummaa ilaalchisee** | | | |
| 25 | Erga deessee turtii yeroo hammami booda Daa’imakee harma hoosifte? | - Yeroodhuma sanatti(01) - Saa’atii ------booda(If <24 hr) (02) - Guyyaa---------booda(03) - Hin yaadadhu(04) |  |
| 26 | Daa’ima keessan guyyaa jalqabaa harma otoo hin hoosisiin dura nyaata/dhangala’aa kennitaniifii turtanii? | - Eyyee (01) - Miti?lakki (02) | Yoo miti ta’e gara G-28darbi |
| 27 | Yoo kennittaniif ta’e maal keennitaniif? | - Bishaan(01) - Dadhaa(02) - Aannan(03) - Kan biro------------------(04) |  |
| 28 | Annan Harmaa kan jalqaba bahe elmitanii gattanii? | - Eeyyee(01) - Miti/lakki(02) | Yoo miti ta’e gara G-30 darbi |
| 29 | Yoo Eelmitanii gattan ta’e maaliif? | ------------- |  |
| 30 | Ammayyu harma hoosisaa jirtuu? | - Eeyyee(01) - Miti/lakki(02) | Eeyyee yoo ta’e gara G-32 ti darbi |
| 31 | Yoo miti ta’e maaliif? | -------- |  |
| 32 | Sa’aatii 24 darban kana keessatti yeroo meeqa hoosiftan? | -------------------- |  |
| 33 | Sa’aatii 24 darban kana keessatti harma irratti dabalataan nyaata /dhangal’aa kennitaniifii turtanii? | - Eeyyee(01) - Miti/lakki(02) | Yoo miti ta’e gaaffii 34 fi 35 dhiisaa. |
| 34 | Yoo eeyyee ta’e maalfaa kennitaniif? | - Aannan sa’aa(01) - Dhadhaa(02) - Shayee, Bulbulamaa Sukkaaraa(03) - Daakuu aannanii Gabaa irraa bitamu(04) - Shoorbaa(axmiet) (05) - Kan biraa(ibsi)------------------(06) |  |
| 35 | Nyaata dabalataa kana daa’ima keessaniif keennuu maaliif barbaaddani | ---------------------------- |  |
